# Effects of statins on serum sCD40, sCD146 and PAPP-An in patients with coronary heart disease

**DOI:** 10.5937/jomb0-55833

**Published:** 2025-08-21

**Authors:** Huawen Li, Xia Zhou, Wenyan Zhu, Jing Zhang

**Affiliations:** 1 South the Taihu Lake Hospital, Huzhou University, Department of Cardiology, Zhejiang Province, China; 2 Zhoushan Branch of the Affiliated Hospital of Shanghai Jiao Tong University School of Medicine, Department of Cardiology, Huzhou, China

**Keywords:** serum sCD40, sCD146, PAPP-An, statins, coronary heart disease, serum factor, serumski sCD40, sCD146, PAPP-An, statini, koronarna bolest srca, serumski faktori

## Abstract

**Background:**

To analyse the effects of statins on the levels of sCD40, sCD146 and PAPP-An in patients with coronary heart disease (CHD).

**Methods:**

126 patients with CHD treated from September 2022 to September 2024 were divided into a study group (n = 84) and a control group (n=42). The study group was randomly divided into groups A (n=42) and B (n=42). Patients in the control group were routinely treated with vasodilator, furosemide, digitalis and other cardiotonic agents. Based on routine treatment, patients in the study group were treated with Atto vastatin calcium tablets in group An and rosuvastatin calcium tablets in group B. Both groups were treated with 20 mg, oral administration before bedtime, and the treatment cycle was 3 months. The cardiac function grade, blood lipid level, serum sCD40, sCD146 and PAPP-A levels and adverse reactions were compared before and after treatment.

**Results:**

After treatment, the cardiac function of the three groups improved compared with that before treatment. The effect of patients in groups A and B was better than the control group. The blood lipid levels of the three groups were improved after treatment. The blood lipid levels of groups A and B were better than in the control group. The serum sCD40, sCD146, and PAPP-A levels between groups A and B were no different; after treatment, these in groups A and B were better than those in the control group. After treatment, there were some adverse reactions in all the three groups.

**Conclusions:**

Statins effectively treat sCD40, sCD146, and PAPP-An in patients with CHD. They can significantly improve their cardiac function and blood lipid levels and effectively regulate sCD40, sCD146, and PAPP-An levels in patients with coronary heart disease

## Abstract (sr)

**Uvod:**

Analiza efekata statina na nivoe sCD40, sCD146 i PAPP-An kod pacijenata sa koronarnom bolešću srca (CHD).

**Metode:**

U studiju je uključeno 126 pacijenata sa CHD lečenih u periodu od septembra 2022. do septembra 2024. Pacijenti su podeljeni u studijsku grupu (n = 84) i kontrolnu grupu (n=42). Studijska grupa je dalje nasumično podeljena u grupu A (n=42) i grupu B (n=42). Pacijenti u kontrolnoj grupi su lečeni standardnom terapijom, uključujući vazodilatatore, furosemid, digitalis i druge kardiotonike. Pacijenti u studijskoj grupi su, uz standardnu terapiju, dobijali atorvastatin kalcijum tablete (grupa A) ili rozuvastatin kalcijum tablete (grupa B). Oba leka su primenjivana oralno, u dozi od 20 mg pre spavanja, tokom 3 meseca. Upoređeni su stepen srcane funkcije, nivo lipida u krvi, nivoi serumskih biomarkera sCD40, sCD146 i PAPP-A, kao i neželjene reakcije pre i nakon terapije.

**Rezultati:**

Nakon terapije, srcana funkcija u sve tri grupe se poboljšala u odnosu na stanje pre lečenja. Poboljšanje je bilo izraženije u grupama A i B u poređenju sa kontrolnom grupom. Nivoi lipida u krvi takođe su poboljšani u sve tri grupe, pri čemu su rezultati u grupama A i B bili bolji nego u kontrolnoj grupi. Nivoi serumskih sCD40, sCD146 i PAPP-A nisu pokazali značajnu razliku između grupa A i B, ali su u obe grupe bili značajno bolji nego u kontrolnoj grupi. Nakon terapije, u sve tri grupe su zabeležene određene neželjene reakcije.

**Zaključak:**

Statini su efikasni u terapiji pacijenata sa CHD, značajno poboljšavaju srčanu funkciju i nivo lipida u krvi, te efikasno regulišu nivoe sCD40, sCD146 i PAPP-A kod pacijenata sa koronarnom bolešću srca.

## Introduction

Coronary atherosclerotic visceral disease (CAVD) is referred to as coronary heart disease (CHD) [1]. The cause of CHD is closely related to hypercholesterolemia, hypertension, diabetes, smoking and obesity. Dyslipidemia is one of the main causes of CHD [2]. Recent projections indicate that by 2050, over 50% of U.S. adults are expected to have hypertension or obesity, with rates exceeding 80% in certain groups, such as Black and older adults. These trends underscore the critical need for effective prevention and management strategies to address the growing burden of cardiovascular disease [3].

The long-term high-oil and high-sugar diet can easily cause hyperlipidemia and hypertension, which greatly increases the incidence of CHD. When the coronary artery blood supply and myocardial oxygen demand are unbalanced, the coronary artery cannot meet the blood flow needed in the process of myocardial metabolism, resulting in myocardial ischemia (MI) and hypoxia [4]. Short-term MI and hypoxia can cause angina pectoris, and continuous MI and hypoxia can cause myocardial infarction and other serious effects [5]. According to statistics, the incidence of chronic heart failure in patients with CHD is increasing year by year, which has many effects on patients' bodies and daily life. Vasodilators, diuretics and cardiotonic agents are commonly used in the clinical treatment of patients with chronic heart failure of CHD [6]. However, the effect of routine clinical treatment is not obvious in most elderly patients because of their weak physique or combined with other systemic diseases. Clinical treatment found that the effective control of serum levels of sCD40, sCD146 and PAPP-An is beneficial to the treatment and prevention of CHD. Statins have sound anti-inflammatory effects, reduce cholesterol, improve endothelial function, and accelerate the shedding of smooth muscle cells in blood vessels [7]. However, the effect of statins on serum sCD40, sCD146 and PAPP-A levels in patients with CHD is not apparent. In this study, different statins were used to treat CHD and to observe their effects on cardiac function, serum sCD40, sCD146, PAPP-An and blood lipids in patients with CHD.

## Materials and methods

### General information

126 CHD patients treated in our hospital from September 2022 to September 2024 were included in this study. The admission criteria were as follows: (1) Coronary angiography showed coronary artery stenosis and diagnosed as CHD (CHD); (2) ECG showed elevation of ST broken arch and pathological Q wave; (3) patients and their family members informed consent and cooperation with treatment; (4) after admission, the patients were graded according to cardiac function, ranging from grade II to grade IV.

Exclusion criteria: (1) patients with severe heart failure, (2) patients with severe liver insufficiency such as active liver disease, patients with severe renal insufficiency, (3) patients with serious mental or nervous system diseases, (4) patients with allergy to drugs used in this study, (5) patients with consumptive diseases such as a malignant tumour, pulmonary tuberculosis, hyperthyroidism, etc.

### Methods

After admission, the patient's health was evaluated, the amount of drinking water was controlled, and the blood sugar and blood pressure of the patients were controlled. Patients are on a light diet, and greasy and spicy diets are prohibited. During treatment, all drugs except prescription drugs given by doctors are not permitted.

The control group patients received standard treatment, such as vasodilator, furosemide and other diuretics, digitalis and other cardiotonic agents. Digitalis preparation was used to closely detect the ECG and observe whether the patients had any adverse reactions. The patient was given aspirin 300 g of orally on the first day of admission, 100 mg on the second day, 5000 U of low molecular weight heparin sodium for two consecutive days, adjacent to the umbilical cord, subcutaneously, 10 mg nitroglycerin was added to 50 mL saline and pumped through a micro pump. The anti-ischemic drugs were treated with clopidogrel combined with antiplatelet therapy.

Based on routine treatment, patients in group A were treated with Atto vastatin calcium tablets (specification: 20 mg/tablets, manufacturer: Lepu Pharmaceutical Technology Co., Ltd., Chinese medicine: H20163270), dosage: 20 mg/, taking time: oral before going to bed, treatment cycle: 3 months. Group B was treated with rosuvastatin calcium tablets (specification: 5 mg/tablets, manufacturer: Lunan Beit Pharmaceutical Co., Ltd., national medicine standard: H20080240), dosage: 20 mg/times, taking time: oral before going to bed, treatment period: 3 months.

### Observation index

The cardiac function grading of patients was assessed using the New York Heart Association (NYHA) classification system. This system categorises patients into four grades (I-IV) based on their physical activity limitations and symptoms of heart failure. Grade I represents patients with no significant limitations in physical activity, while Grade IV includes patients with severe limitations and symptoms at rest. The classification was used to evaluate the improvement in cardiac function before and after treatment.

The blood lipid profile was measured to assess changes in lipid metabolism and cardiovascular risk. Total cholesterol (TC) and triglycerides (TG) levels were recorded using an automatic biochemical analyser. Low-density lipoprotein cholesterol (LDL-C) was measured as a major risk factor for atherosclerosis and coronary heart disease, while high-density lipoprotein cholesterol (HDL-C) was evaluated as it plays a protective role against cardiovascular disease. The changes in these lipid parameters after treatment were compared among the groups.

The serum biomarkers of sCD40, sCD146, and PAPP-A were measured using enzyme-linked immunosorbent assay (ELISA). Soluble CD40 ligand (sCD40) is an inflammatory marker that plays a role in the progression of atherosclerosis and plaque instability. Soluble CD146 (sCD146) is an endothelial adhesion molecule associated with angiogenesis and neovascularisation, which can influence vascular integrity in CHD. Pregnancy-associated plasma protein-A (PAPP-A) is an important marker of plaque rupture and inflammation, with elevated levels indicating higher cardiovascular risk. The effect of statin treatment on these biomarkers was evaluated in the study.

The adverse reactions observed during the study included headache, nausea, abdominal pain, diarrhoea, and constipation. The frequency and severity of these adverse effects were recorded in all three groups. A comparison was made to determine whether statin therapy led to fewer adverse reactions than conventional treatment. Monitoring of side effects helped assess the safety profile of atorvastatin and rosuvastatin in CHD patients over the three-month treatment period.

All parameters were measured at baseline and after three months of treatment. An automatic biochemical analyser was used for lipid level assessment, and ELISA was employed to measure serum biomarker levels. The data collected from these evaluations were analysed to determine the effectiveness of statins in improving cardiac function, lipid metabolism, and inflammatory markers in patients with coronary heart disease.

### Statistical method

The SPSS20.0 software package statistically analysed the research data; the measurement data were expressed as standard deviation (x̄±s), the F test was used for inter-group comparison, the LSD-t test was used for pairwise comparison, counting data was expressed as [n (%)], χ^2^ [test was used for inter-group comparison, and Ridit analysis was used for rank data (ordered classification variables).

## Results

There were 68 males and 58 females aged 53 to 72 years, with an average of (63.87±6.52) years. The course of disease was 2-6 years. The group was randomly divided into a study group (n = 84) and a control group (n = 42). The study group was randomly divided into groups A (n = 42) and B (n=42). The gender, age, disease course and other diseases among the three groups were no different (Table 1).

**Table 1:** General data between two groups of patients.

Group	Study group		Control group<br>(n=42)	F/χ^2^	P
	A (n=42)	B (n=42)			
Gender	21/18	23/20	24/20	0.343	0.842
Age	61.93±7.16	62.71±6.87	62.24±6.41	0.14	0.870
Course of disease	4.13±1.54	4.03±1.87	4.61±1.42	1.54	0.219
Complicated with diabetes mellitus	3 (7.14%)	3 (7.14%)	2 (4.76%)	0.266	0.875
Complicated with hypertension	6 (14.28%)	8 (19.04%)	7 (16.66%)	0.342	0.842
Complicated with cerebrovascular disease	4 (9.52%)	3 (7.14%)	5 (11.90%)	0.552	0.758

### The cardiac function grades of the three groups

The cardiac function grades between groups A and B were no different before treatment. After treatment, the patients in the three groups were improved. The effect of patients in groups A and B was better than in the control group (Table 2).

**Table 2:** Comparison of cardiac function grades among the three groups.

Group		N	NYHA grading before treatment				NYHA grading after treatment			
			I	II	III	IV	I	II	III	IV
Study<br>group	A	42	1	15	24	2	25	13	4	0
	B	42	0	15	24	3	23	16	3	0
Control group		42	0	17	23	2	13	16	12	1
χ^2^						0.245				9.702
P						0.885				0.008

### The levels of blood lipids in the three groups compared

Before treatment, the blood lipid levels of study groups A and B were the same. After treatment, however, the blood lipid levels of the three groups improved. After treatment, the blood lipid levels of groups A and B were better than those of the control group (Table 3).

**Table 3:** Compare the blood lipid levels of the three groups.

Group		N	Total cholesterol		Triglyceride		Low-density lipoprotein<br>cholesterol		High-density lipoprotein<br>cholesterol	
			Before<br>treatment	After<br>treatment	Before<br>treatment	After<br>treatment	Before<br>treatment	After<br>treatment	Before<br>treatment	After<br>treatment
Study<br>group		42	5.32±0.54	4.09±0.51	1.98±0.62	1.42±0.29	3.37±1.39	2.52±0.81	1.08±0.61	1.62±0.56
		42	5.36±0.49	4.12±0.53	1.85±0.72	1.50±0.30	3.47±1.67	2.39±0.90	1.07±0.57	1.69±0.60
t			0.355	0.264	0.886	1.242	0.298	0.695	0.073	0.552
P			0.723	0.791	0.377	0.217	0.766	0.488	0.941	0.581
Control<br>group		42	5.27±0.68	4.77±0.69	2.01±0.61	1.79±0.49	3.41±1.45	3.11±0.62	1.11±0.51	1.33±0.28
t			0.26	18.29	0.71	11.53	0.05	10.03	0.05	6.1
P			0.773	<0.001	0.491	<0.001	0.954	<0.001	0.948	0.003

### The serum levels of sCD40, sCD146 and PAPP-A compared among the three groups

There was no difference in serum levels of sCD40, sCD146, and PAPP-A between groups A and B before treatment. The serum levels of sCD40, sCD146 and PAPP-A between groups A, B and the control group were no different before treatment. After treatment, the serum levels of sCD40, sCD146 and PAPP-A between groups A and B were no different, while those in groups A and B were better than those in the control group (Table 4).

**Table 4:** The serum levels of sCD40, sCD146 and PAPP-A compared among the three group.

Group		N	sCD40 (ng/mL)		sCD146 (ng/mL)		PAPP-A (mU/L)	
			Before<br>treatment	After<br>treatment	Before<br>treatment	After<br>treatment	Before<br>treatment	After<br>treatment
Study<br>group	A	42	13.61±2.43	7.92±2.15	197.38±10.22	183.27±9.82	4.65±0.92	2.31±0.46
	B	42	13.52±2.58	8.05±2.05	201.42±9.24	185.74±9.36	4.38±0.87	2.28±0.61
t			0.164	0.283	1.9	1.799	1.381	0.254
P			0.869	0.777	0.06	0.241	0.17	0.799
Control group		42	13.28±2.38	10.73±2.31	198.64±9.82	192.81±9.86	4.57±0.90	3.77±0.83
t			0.2	22.39	1.88	10.98	1	71.83
P			0.818	<0.001	0.156	<0.001	0.369	<0.001

### The adverse reactions of three groups of patients were compared

After treatment, there were some adverse reactions in all three groups; the adverse reactions between groups A and B were no different. The rate of adverse reactions in the study group was reduced compared to the control group (Table 5).

**Table 5:** The adverse reactions of the three groups were compared.

Group		N	Headache	Nausea	Abdominal pain and diarrhoea	Constipation	Tota
Study group	A	42	1 (2.38%)	2 (4.76%)	0	1 (2.38%)	4 (9.52%)
	B	42	2 (4.76%)	2 (4.76%)	1 (2.38%)	0	5 (11.90%)
Control group	42		2 (4.76%)	3 (7.14%)	4 (9.52%)	3 (7.14%)	12 (28.57)
χ^2^							6.514
P							0.038

## Discussion

As a common clinical cardiovascular disease, CHD occurs mostly in the middle-aged and elderly, with a high mortality and disability rate, which is a serious threat to human health [8]. A small number of patients with CHD have no clinical symptoms; most of them have chest pain, fatigue, palpitation, dyspnea and so on. In severe cases, there will be heart failure, shock, arrhythmia and other symptoms, which need long-term drug treatment. The cause of CHD is closely related to hypercholesterolemia, hypertension, diabetes and obesity caused by long-term high-calorie, high-fat, high-cholesterol and high-sugar diets. Dyslipidemia is one of the main causes of CHD [9] [10].

Hypolipidemic drugs are commonly used in the clinical treatment of CHD. Among the antilipidemic drugs, statins are the most effective reductase inhibitors. Statins produce effects mainly through hydroxymethyl glutaraldehyde-CoA reductase inhibitors. Block the synthesis of cholesterol in the serum to reduce the content of cholesterol in the serum [11] [12]. At the same time, the clinical use of statins can improve vascular endothelial function to a certain extent, improving atherosclerosis and reducing the inflammatory reaction of the vascular wall by blocking endothelial cells and activating adhesion molecules [13].

This study showed that the cardiac function grades between groups A and B were the same before treatment, as were the cardiac function grades between the study and control groups. After treatment, the patients in the three groups improved. The effect of patients in the study group was better than that of the control group.

Statins are suggested to be critical in improving patients' cardiac function. Our study observed that statin therapy significantly improved cardiac function and lipid profiles in coronary heart disease (CHD) patients. These findings align with previous research demonstrating that statins lower lipid levels and exert anti-inflammatory and plaque-stabilising effects. For instance, a meta-analysis by Nissen et al. [14] concluded that intensive statin therapy slows atherosclerotic plaque progression and may lead to plaque regression.

Furthermore, our study found that statin treatment effectively reduced serum levels of sCD40, sCD146, and PAPP-A, biomarkers associated with inflammation and endothelial dysfunction in CHD patients. This is consistent with the pleiotropic effects of statins, including anti-inflammatory properties and endothelial function improvement. Morofuji et al. [15] discussed these additional benefits of statins, highlighting their role in cardiovascular protection beyond lipid-lowering.

The severity of CHD is closely related to the levels of sCD40, sCD146 and PAPp-An in serum [16] [17]. SCD146 is a vascular endothelial cell adhesion factor that can promote the signal transduction of the VEGF receptor and the formation of the vascular structure of endothelial cells. It can induce neovascularisation in CHD. When neovascularisation occurs, it leads to internal bleeding and thrombus [18]. SCD40 can aggravate the inflammatory response in the body, leading to plaque instability in patients and a vicious circle [19]. PAPP-An is mainly distributed in macrophages and fibroblasts, and many of PAPP-An exist in ruptured plaques. When the concentration of PAPP-An in serum increases, it represents the development of a chronic inflammatory reaction, which leads to the aggravation of the disease [20]. When the levels of sCD40, sCD146 and PAPP-A rise, it will aggravate the inflammatory reaction in the patient's body, make the plaque in the blood vessel unstable, and more seriously lead to plaque rupture, bleeding and so on. The serum levels of sCD40, sCD146, and PAPP-An among the three groups showed no difference before treatment. After treatment, those in the study group were better than the control group. Additionally, the reduction in adverse reactions observed in our study's statin-treated groups compared to the control group underscores the safety and tolerability of statin therapy in CHD management. This supports the broader clinical evidence that statins are generally well-tolerated and effective in reducing cardiovascular events.

To sum up, statins effectively treat sCD40, sCD146 and PAPP-An in patients with CHD. They can significantly improve cardiac function and blood lipid levels, reduce the levels of sCD40, sCD146 and PAPP-An in patients with CHD, and the incidence of adverse reactions is less than that of conventional treatment.

## Dodatak

### Conflict of interest statement

All the authors declare that they have no conflict of interest in this work.
